# Impact of ^18^F-FDG PET/CT, CT and EBUS/TBNA on preoperative mediastinal nodal staging of NSCLC

**DOI:** 10.1186/s12880-021-00580-w

**Published:** 2021-03-17

**Authors:** Akram Al-Ibraheem, Nader Hirmas, Stefano Fanti, Diana Paez, Fawzi Abuhijla, Dalia Al-Rimawi, Ula Al-Rasheed, Riad Abdeljalil, Feras Hawari, Kamal Alrabi, Asem Mansour

**Affiliations:** 1 Department of Nuclear Medicine, King Hussein Cancer Center, Queen Rania Al-Abdullah Street 202, P.O. Box 1269, Amman, Jordan; 2 Nuclear Medicine Clinic, Essen University Hospital, Hufelandstrasse 55, 45147 Essen, Germany; 3 Department of Nuclear Medicine, Policlinico S. Orsola, Università di Bologna, Bologna, Italy; 4 Nuclear Medicine and Diagnostic Imaging Section, Division of Human Health, International Atomic Energy Agency, Vienna, Austria; 5 Department of Radiation Oncology, King Hussein Cancer Center, Queen Rania Al-Abdullah Street 202, Amman, Jordan; 6 Office of Scientific and Academic Research (OSAR), King Hussein Cancer Center, Queen Rania Al-Abdullah Street 202, Amman, Jordan; 7 Department of Surgery, King Hussein Cancer Center, Queen Rania Al-Abdullah Street 202, P.O. Box 1269, Amman, Jordan; 8 Department of Medicine, Section of Pulmonary and Critical Care, King Hussein Cancer Center, Queen Rania Al-Abdullah Street 202, P.O. Box 1269, Amman, Jordan; 9 Department of Internal Medicine, Hematology and Oncology, King Hussein Cancer Center, Queen Rania Al-Abdullah Street 202, P.O. Box 1269, Amman, Jordan; 10 Department of Diagnostic Radiology, King Hussein Cancer Center, Queen Rania Al-Abdullah Street 202, P.O. Box 1269, Amman, Jordan

**Keywords:** NSCLC, ^18^F-FDG PET/CT, EBUS/TBNA, Mediastinal lymph node staging, NPV

## Abstract

**Background:**

Staging of non-small-cell lung cancer (NSCLC) is a multidisciplinary process involving imaging, endoscopic and surgical techniques. This study aims at investigating the diagnostic accuracy of ^18^F-FDG PET/CT, CT scan, and endobronchial ultrasound/transbronchial needle aspirate (EBUS/TBNA) in preoperative mediastinal lymph nodes (MLNs) staging of NSCLC.

**Methods:**

We identified all patients who were diagnosed with NSCLC at the King Hussein Cancer Center in Amman, Jordan, between July 2011 and December 2017. We collected their relevant clinical, radiological, and histopathological findings. The per-patient analysis was performed on all patients (N = 101) and then on those with histopathological confirmation (N = 57), followed by a per-lymph-node-station basis overall, and then according to distinct N-stage categories.

**Results:**

^18^F-FDG PET/CT, in comparison to CT, had a better sensitivity (90.5% vs. 75%, *p* = 0.04) overall and in patients with histopathological confirmation (83.3% vs. 54.6%), and better specificity (60.5% vs. 43.6%, *p* = 0.01) overall and in patients with histopathological confirmation in MLN staging (60.6% vs. 38.2%). Negative predictive value of mediastinoscopy, EBUS/TBNA, and ^18^F-FDG PET/CT were (87.1%), (90.91%), and (83.33%) respectively. The overall accuracy was highest for mediastinoscopy (88.6%) and EBUS/TBNA (88.2%), followed by ^18^F-FDG PET/CT (70.2%). Dividing patients into N1 disease vs. those with N2/N3 disease yielded similar findings. Comparison between ^18^F-FDG PET/CT and EBUS/TBNA in patients with histopathological confirmation shows 28 correlated true positive and true negative findings with final N-staging. In four patients, ^18^F-FDG PET/CT detected metastatic MLNs that would have otherwise remained undiscovered by EBUS/TBNA alone. Lymph nodes with a maximal standardized uptake value (SUVmax) more than 3 were significantly more likely to be true-positive.

**Conclusion:**

Multimodality staging of the MLNs in NSCLC is essential to provide accurate staging and the appropriate treatment. ^18^F-FDG PET/CT has better overall diagnostic utility when compared to the CT scan. The NPV of ^18^F-FDG PET/CT in MLNs is reliable and comparable to the NPV of EBUS/TBNA. SUVmax of MLNs can help in predicting metastases, but nevertheless, a positive ^18^F-FDG PET/CT MLNs particularly if such a result would change the treatment plan, should be verified histopathologically.

**Supplementary Information:**

The online version contains supplementary material available at 10.1186/s12880-021-00580-w.

## Background

Staging of non-small-cell lung cancer (NSCLC) is a multidisciplinary process involving imaging, endoscopic and surgical techniques. Accurate clinical staging of mediastinal lymph nodes (MLNs) is crucial for selecting candidate patients for surgical resection. The involvement of MLNs is a very important prognostic factor in patients with potentially resectable NSCLC. Stage I, II, and III patients with no lymph node metastases (N0) or ipsilateral hilar lymph metastases (N1) stage are usually referred to surgical resection, whereas patients with ipsilateral mediastinal lymph metastases (N2) or contralateral mediastinal lymph metastases (N3) disease are referred to chemoradiation. [1, 2].

Accuracy is vital in order to avoid false-positive interpretations leading to a false stage III diagnosis in early-stage patients, or false-negative findings leading to a false early-stage diagnosis in patients with MLNs disease. Mediastinoscopy has been traditionally considered the gold standard for mediastinal staging to assess potential N2 and N3 nodal involvement. Given it is an invasive diagnostic procedure, a small risk of complications exists, such as pneumothorax, recurrent laryngeal nerve injury, hemorrhage, and tracheal laceration [3]. In addition, conventional mediastinoscopy would not have access to all stations, as it normally surveys LN stations 2, 4, and 7 only.

Computed tomography (CT) scan offers an excellent anatomical detail of tumor spread, but radiological imaging lacks information on the biological nature of the lesions. The latter is brought in by 2-[fluorine-18] fluoro-2-deoxy-D-glucose positron emission tomography (^18^F-FDG PET) scan as a metabolic imaging tool, which, however, has clearly lower spatial resolution. Therefore, contemporary staging relies on the combination of both, using a PET/CT scan [4].

By evaluating the detection accuracy of CT scan alone, ^18^F-FDG PET/CT, and other invasive procedures, we can determine if the negative predictive value of ^18^F-FDG PET/CT is sufficient on its own in guiding management of patients with NSCLC, perhaps without further subjecting them to further invasive procedures.

This retrospective cohort study aims at investigating the diagnostic accuracy of ^18^F-FDG PET/CT, CT scan, and endobronchial ultrasound/transbronchial needle aspirate (EBUS/TBNA) in preoperative MLN staging of NSCLC in comparison to mediastinoscopy and histopathological diagnosis.

## Methods

### Study population and data analysis

This is a retrospective cohort study. Between July 2011 and December 2017, a total of 145 patients received ^18^F-FDG PET/CT for the staging of NSCLC after confirmed tissue diagnosis. All patients gave informed consent to undergo ^18^F-FDG PET/CT. This study was approved by the Institutional Review Board (IRB) at the King Hussein Cancer Center (KHCC) in Amman, Jordan. After going through patients’ online medical records, patients who have pathological confirmation of NSCLC, with their data available in our records, and without any prior surgical treatments were included in this study. Patients with stage IV disease without proper nodal staging assessments were excluded since there is no objective assessment of their nodal staging status. Subsequently, 101 patients (70%) were included in the final analysis.

Using a pre-designed case report form, we retrieved the following information: patient demographics; clinical and histopathological findings; radiological findings for CT and ^18^F-FDG PET/CT stratified according to T, N, and M categories; pathological findings through EBUS/TBNA and mediastinoscopy; and medical and surgical treatment options offered.

The diagnostic accuracies of CT, ^18^F-FDG PET/CT, and EBUS/TBNA were then analyzed in relation to histopathological diagnosis by comparing to findings of mediastinoscopy or lymph node dissection whenever performed, multidisciplinary clinic (MDC) decisions at our center as well as any follow-up radiological and clinical data. This analysis was first performed on a per-patient basis (N = 101), where each diagnostic modality was assessed in relation to the final correct nodal staging of each patient. The subsequent detailed analysis included patients with histopathological diagnosis (N = 57) and compared all these diagnostic modalities on a per-patient basis, followed by a per-lymph-node-station basis (for LN stations 2, 4, 5, 7, 9, and 10, where appropriate) overall, and then according to distinct N-stage categories (N0, N1, and N2/3). Only one patient with a confirmed histopathological diagnosis had N3 staging and was included in the N2 staging category for easier analysis.

### ^18^F-FDG PET/CT imaging

^18^F-FDG PET/CT was performed using a Biograph mCT PET/CT machine (Siemens Medical Solutions, Erlanger, Germany). The procedure followed was the one recommended by the European Association of Nuclear Medicine [5]. All patients fasted for at least 6 h. It was verified that their blood glucose levels were below 200 mg/dL before the administration of ^18^F-FDG. The ^18^F-FDG dose administered was 3 MBq/Kg. After injection of ^18^F-FDG, patients remained at rest for around 60 min in a room prepared to this end. Body CT study was obtained from the base of the skull to the middle of the thigh in a cranial-caudal direction using a free-breathing respiration signal. No IV contrast was given. Finally, the PET was performed on the same locations in a caudal-cranial, in free respiration.

Conventional contrast-enhanced CT scans (CE-CT) and ^18^F-FDG PET/CT scans were performed within 11 days of each other. CT results were obtained by recording the reports from the online medical records as interpreted by a radiologist at KHCC. CT-positive nodes were defined by increased short-axis diameter (more than 8 mm), loss of fatty hilum, or increased contrast enhancement.^18^F-FDG PET/CT scans were interpreted by a consensus read of two nuclear medicine physicians. Maximum intensity projection (MIP) images were evaluated with different intensity scales, after which images were displayed side-by-side. Axial, sagittal, and coronal PET reconstruction were interpreted with and without attenuation correction. Corresponding CT images were also acquired in a Biograph mCT flow 64 slices CT, reconstructed in axial, sagittal, and coronal planes, and reviewed alongside the PET images. The location of lesions was determined by the CT component of the study. Patients’ weights were measured routinely before IV administration of radioisotope.

PET-positive LNs were identified by ^18^F-FDG uptake visually above mediastinal physiologic blood pool background activity and not associated with any physiologic uptake. The implemented intensity-scale bar of PET images was the maximal standardized uptake value (SUVmax). SUVs were calculated according to the formula: SUV = measured activity within the region of interest (MBq/mL)/[injected dose of FDG (MBq)/body weight (g). SUVmax of the tumor and LNs were calculated from regions drawn manually over sites of most intense increased uptake. The windowing of the CT images was within the range of the soft tissue windowing W:350 L:50 in HU. This has been added to the methodology section.

Real time Endobronchial ultrasound-guided transbronchial needle aspiration (EBUS-TBNA) of lymph nodes was performed within 20 days interval of CE-CT and ^18^F-FDG PET/CT under conscious sedation using a variable combination of fentanyl, midazolam, and propofol. A single operator determined the Lymph node stations that should be sampled based on the accessible station that was harboring a sizable and considerably hypermetabolic lymph node, mainly based on the PET/CT findings. Using a flexible bronchoscope with an integrated linear ultrasound transducer, at least 3 passes were made in each lymph node station with a 21 gauge TBNA needle. Rapid on-site evaluation was performed when available (ROSE). The collected sample was injected into collecting tubes filled with CytoLyte (methanol–water solution) and sent to the lab for further processing.

### Surgical-pathological staging

All procedures and surgeries were performed by the same team of thoracic surgeons at KHCC within 32 days of the diagnostic CE-CT and ^18^F-FDG PET/CT. Systematic mediastinal lymph node dissections were performed in appropriate cases based on prior MDC decisions. The mediastinal and hilar lymph nodes removed were tagged and sent to the anatomic pathology service at KHCC separately in lymph node stations, based on the lymph node map proposed by the International Association for the Study of Lung Cancer [6]. Also, the intrapulmonary lymph nodes (stations 12, 13, and 14) were removed with the surgical piece, where appropriate. All tumors resected were examined by experienced pathologists in lung examination who did not know the results from the ^18^F-FDG PET/CT.

### Statistical analysis

Patients’ characteristics were presented as counts and percentages for categorical variables, such as gender and stage, while the median and range were used for continuous variables. Confusion matrix was used to find predication values including sensitivity, specificity, negative predictive value (NPV), and positive predictive value (PPV). Additionally, corresponding, exact binomial 95% confidence intervals were calculated. McNemar’s test was used to compare diagnostic abilities between each two imaging techniques in terms of sensitivity and specificity. Comparisons between two imaging techniques' TP and TN values were performed using Chi square or Fisher's exact test as appropriate A significance criterion of *p* ≤ 0.05 was used in the analysis. All analyses were performed using SAS version 9.4 (SAS Institute Inc., Cary, NC).

## Results

### Patient and tumor characteristics

A total of 101 patients were included in the study (Table 1), with a M: F ratio of 4.6:1. The median age at diagnosis was 62 years (range 29.8–77.7), with 74.3% being smokers or previous smokers at the time of diagnosis. The most common histopathology was adenocarcinoma (56.4%), followed by squamous cell carcinoma (37.6%). Median FDG-positive tumor size was 5.5 cm (range 1.3–15 cm) with a median SUVmax tumor of 11 (range 2.7–36) and median SUVmax lymph node of 5 (range 1.5–19.5). Right upper lobe (RUL) was the most involved lobe in 43.6% followed by left upper lobe (LUL) in 30.7%. Stage III was the most common stage (58.4%) followed by stages II and I (15.8% each). ^18^F-FDG PET/CT was performed in all patients, CT scan was done in 98% of cases, EBUS/TBNA in 39.6%, and mediastinoscopy in 40.6%. More than half of the patients underwent surgical lymph node dissection or biopsy (56.4%).

**Table 1:** Patient characteristics

Characteristics	N (%)
No. of patients	101
Male	83 (82)
Female	18 (18)
Median age, years (range)	62.0 (29.8–77.7)
Smoking history	
Current smoker	53 (52.5)
Any history of smoking	75 (74.3)
Histopathology	
Adenocarcinoma	57 (56.4)
Adenosquamous carcinoma	4 (4)
Squamous cell carcinoma	38 (37.6)
Undifferentiated carcinoma	2 (2)
Median tumor size, cm (range)	5.5 (1.3–15)
Median SUVmax tumor (range)	11 (2.7–36)
Median SUVmax lymph nodes (range)	5 (1.5–19.5)
Lobar involvement^a^	
RUL	44 (43.6)
RML	14 (13.9)
RLL	9 (8.9)
LUL	31 (30.7)
LLL	11 (10.9)
Clinical stage	
I	16 (15.8)
II	16 (15.8)
III	59 (58.4)
IV	10 (9.9)
Scans/procedures performed	
^18^F-FDG PET/CT	101 (100)
CT scan	99 (98)
EBUS/TBNA	40 (39.6)
Mediastinoscopy	41 (40.6)
Lymph node biopsy or dissection	57 (56.4)

^a^May involve multiple lobes

### Per-patient analysis

A per-patient analysis for concordance with N-staging was performed on all 101 patients included in the study (Table 2). In comparison to CT, ^18^F-FDG PET/CT had a better sensitivity (90.5% vs. 75%, *p* = 0.04), specificity (60.5% vs. 43.6%, *p* = 0.01), PPV (79.2% vs. 67.2%), NPV (79.3% vs. 53.1%) and accuracy (79.2% vs. 62.6%). Sensitivity of ^18^F-FDG PET/CT (90.5%, 95% CI 83.2–97.7%) was comparable to that of EBUS/TBNA (84.6%, 95% CI 70.7–98.5%, *p* = 0.13) and higher than mediastinoscopy (66.7%, 95% CI 40.0–93.3%, *p* = 0.133). The overall accuracy for mediastinoscopy was highest (90.2%), compared to EBUS/TBNA (87.5%), ^18^F-FDG PET/CT (79.2%) and CT scan (63.6%).

**Table 2:** Diagnostic utility of the different imaging modalities (per-patient analysis—ALL)

Imaging modality	Sensitivity (%) 95% CI	Specificity (%) 95% CI	PPV (%) 95% CI	NPV (%) 95% CI	Accuracy (%) 95% CI
^18^F-FDG PET/CT	90.5 80.41–96.42	60.5 43.39–75.96	79.2 71.77–85.03	79.3 63.19–89.54	79.2 69.99–86.64
CT scan	75 62.14–85.28	43.6 27.81–60.38	67.2 59.95–73.65	53.1 39.17–66.61	62.6 52.33–72.15
EBUS/TBNA	84.6 65.13–95.64	92.9 66.13–99.82	95.7 76.77–99.32	76.5 56.60–89.01	87.5 73.20–95.81
Mediastinoscopy	66.7 34.89–90.08	100 88.06–100.00	100 NA	87.9 76.51–94.16	90.2 76.87–97.28

Another round of per-patient analysis for concordance with N-staging was performed on 57 patients with confirmed histopathological diagnosis (Table 3). ^18^F-FDG PET/CT, in comparison to CT, had a better sensitivity (83.3% vs. 54.6%, *p* = 0.18) and better specificity (60.6% vs. 38.2%, *p* = 0.01). The ensitivity of ^18^F-FDG PET/CT matched that of EBUS/TBNA (83.3%) and was higher than that of mediastinoscopy (50%). The overall accuracy was highest for mediastinoscopy (88.6%) and EBUS/TBNA (88.2%), followed by ^18^F-FDG PET/CT (70.2%).

**Table 3:** Diagnostic utility of the different imaging modalities (per-patient analysis—histopathology available)

Imaging modality	Sensitivity (%) 95% CI	Specificity (%) 95% CI	PPV (%) 95% CI	NPV (%) 95% CI	Accuracy (%) 95% CI
^18^F-FDG PET/CT	83.3 62.62–95.26	60.6 42.14–77.09	60.6 49.28–70.89	83.3 66.23–92.73	70.2 56.60–81.57
CT scan	54.6 32.21–75.61	38.2 22.17–56.44	36.4 26.43–47.62	56.5 41.00–70.86	44.6 31.34–58.53
EBUS/TBNA	83.3 35.88–99.58	90.9 58.72–99.77	83.3 42.72–97.10	90.9 62.33–98.37	88.2 63.56–98.54
Mediastinoscopy	50 15.70–84.30	100 87.23–100.00	100 NA	87.1 77.15–93.10	88.6 73.26–96.80

Per-lymph-node-station analysis in the 57 patients with histopathological diagnosis was provided in the Additional file 1: Table S1.

The analysis of the performance of the different modalities according to N-stage categories (N1, N2, and N3) was provided in the Additional file 2.

### ^18^F-FDG PET/CT compared to EBUS/TBNA

**Fig. 1:**
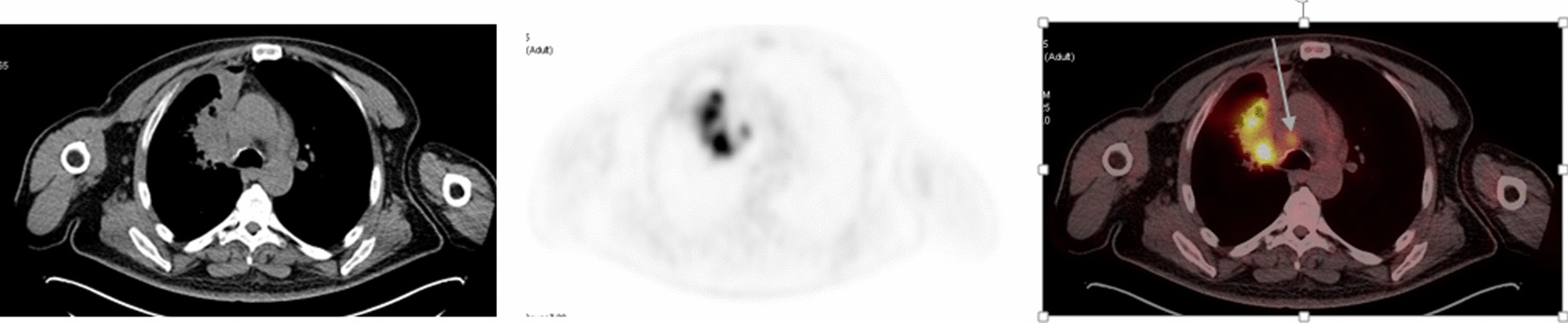
True positive FDG-PET/CT and true positive EBUS/TBNA. A case of moderately differentiated lung adenocarcinoma in the right lung. Axial CT showed prominent pretracheal lymph node. Axial FDG-PET and FDG-PET/CT showed hypermetabolic (SUVmax: 3.8) pretracheal lymph node (arrow) and suggested a metastases. EBUS/TBNA from this lymph node turned positive for metastases that was confirmed later by lymph node dissection

**Fig. 2:**
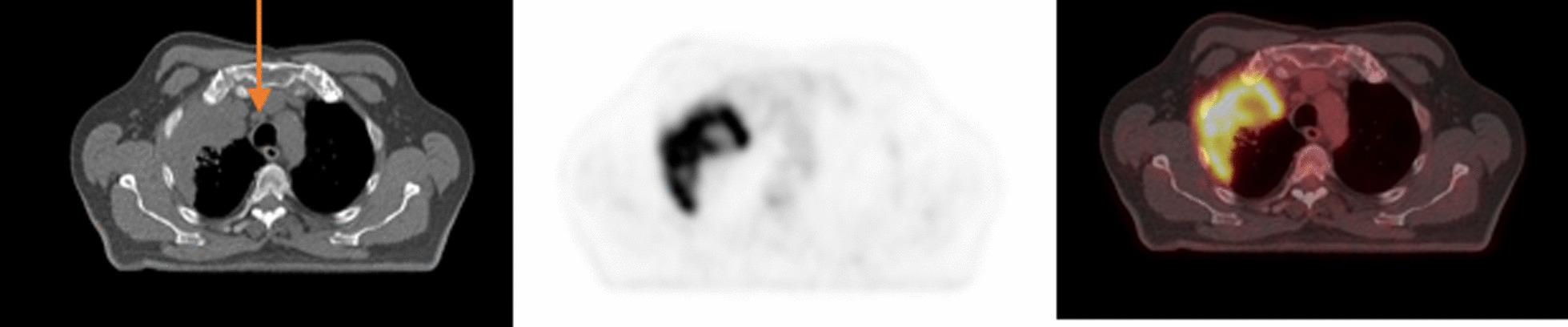
True negative FDG-PET/CT and true negative EBUS/TBNA. A case of poorly differetiated lung adenocarcinoma. Axial CT scan showed prominent right upper paratracheal lymphnode (arrow). Axial FDG-PET and PFDG-PET/CT didn’t show concerning hypermetabolic features (SUVmax: 1.2) in this lymph node. EBUS/TBNA turned negative for metastases in this lymph node that was confirmed during lymph node dissection

**Fig. 3:**
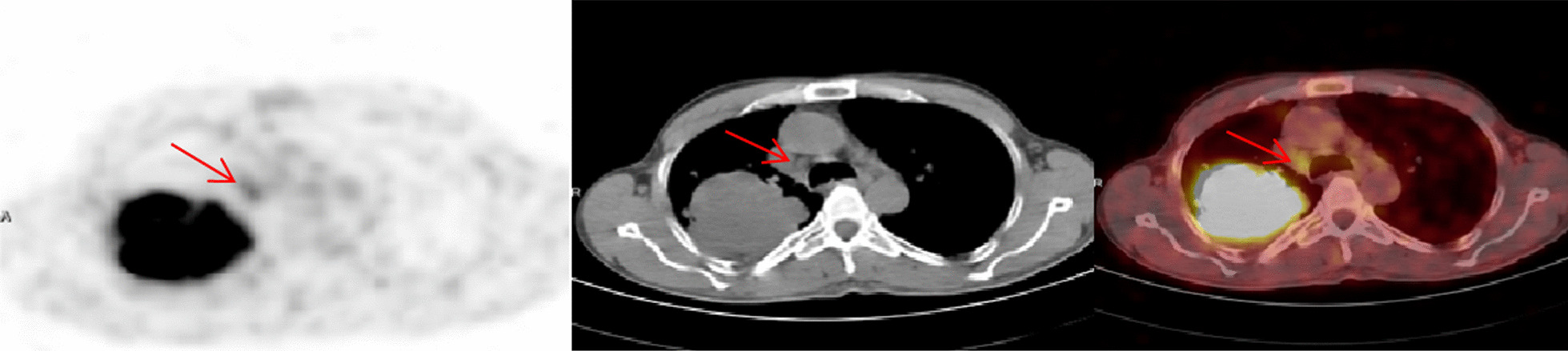
True positive FDG-PET/CT and false negative EBUS/TBNA. A case of SCC involving the right lung. Axial FDG-PET, CT and FDG-PET/CT images showed a suspected prominent hypermetabolic (SUVmax: 3.1) right paratracheal lymph node (arrows) and recommended tissue confirmation. EBUS/TBNA turned out negative; Mediastinoscopy proved a metastatic lymph node

**Fig. 4:**
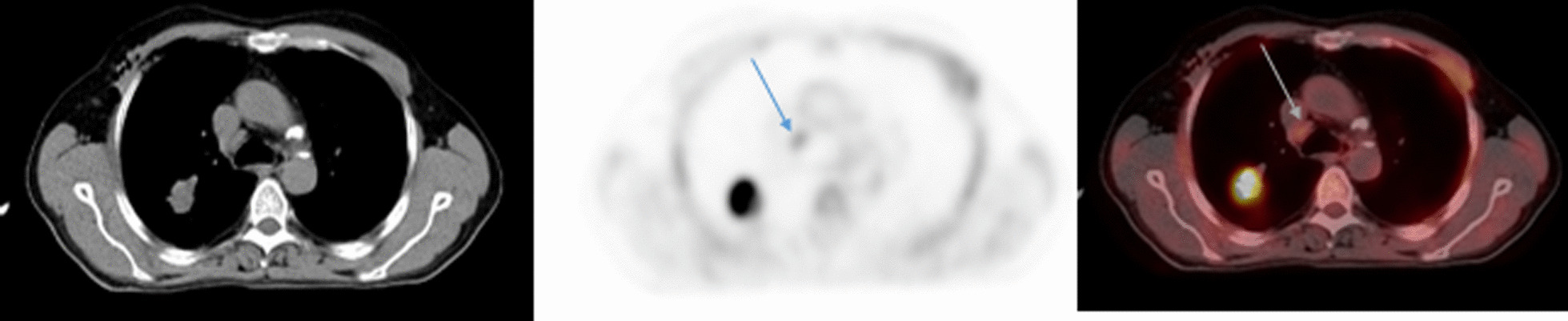
False positive FDG-PET/CT and true negative EBUS/TBNA. A case of right lung moderately differentiated lung adenocarcinoma. Axial CT scan showed prominent right lower paratracheal lymph node. Axial FDG-PET and FDG-PET/CT showed suspicious hypermetabolic (SUVmax: 3) right lower paratracheal lymph node. EBUS/TBNA turned negative for lymph node metastases. Lymph node dissection confirmed the EBUS/TBNA result

Comparison between ^18^F-FDG PET/CT and EBUS/TBNA in patients with histopathological confirmation shows 28 correlated true positive (Fig. 1) and true negative (Fig. 2) findings in concordance with final N-staging. In four patients, ^18^F-FDG PET/CT detected metastatic MLNs that would have otherwise remained undiscovered by EBUS/TBNA alone (Fig. 3). On the other hand, EBUS/TBNA conferred accurate staging in one patient who had a positive sub-carinal LN TBNA result which was missed by ^18^F-FDG PET/CT and would have otherwise been considered N1 stage. Also, EBUS/TBNA showed true negative findings in six cases that were deemed false positive by ^18^F-FDG PET/CT (Fig. 4). Both modalities had correlating one false positive finding. In this subset of patients, ^18^F-FDG PET/CT in comparison to EBUS/TBNA had a higher sensitivity (96.4% vs. 84.6%), lower specificity (41.7% vs. 92.9%), lower PPV (79.4% vs. 95.7%), higher NPV (83.3 vs. 76.5%) and a lower accuracy (80% vs. 87.5%).

### ^18^F-FDG PET/CT and change of management

From the 57 patients with confirmed histopathological diagnosis, ^18^F-FDG PET/CT was successful in achieving concordance with the final N-staging in 40 patients (70%). Of those patients, 10 (25%) underwent a change in management solely on the basis of ^18^F-FDG PET/CT findings as follows: 2 patients were down-staged to N0, 2 patients were down-staged to N1, 1 patient was down-staged to N0, 2 patients were upstaged to N1, 2 patients were upstaged to N2 and 1 patient was upstaged to N3.

Thirteen patients (22.8%) had false-positive findings and would have been incorrectly upstaged by ^18^F-FDG PET/CT if this modality was solely relied upon in clinical management. These were as follows: 4 patients with N0 stage mistaken for N1, 4 patients with N0 stage mistaken for N2, 3 patients with N1 stage mistaken for N2, and 2 patients with N1 stage mistaken for N3.

Four patients (7%) had false-negative findings and would have been incorrectly down-staged by ^18^F-FDG PET/CT if this modality was solely relied upon in clinical management. These were as follows: 2 patients with N2 stage mistaken for N0, and 2 patients with N2 stage mistaken for N1.

### Variables associated with true positivity or negativity in ^18^F-FDG PET/CT

Among 40 patients with true positive and true negative results by 18F-FDG PET/CT, we investigated associations between these and several demographic and clinical variables (Table 4). For tumor location, true negatives were mostly peripherally located (79%), whereas true positives were equally seen centrally and peripherally (50%; *p* = 0.06). In addition, a cut-off SUVmax value of 3 among FDG-avid lymph nodes revealed a significant difference: lymph nodes with SUVmax more than 3 were more likely to be true-positive (*p* = 0.00). Other variables such as the presence of lung disease, diabetes, smoking status, patient’s age, and histopathology of tumor, tumor size or tumor SUVmax were not statistically associated with the distributions of true positives and true negatives.

**Table 4:** Comparing TN and TP findings among different variables in 18F-FDG PET/CT

Variable	Total	TN N (%)	TP N (%)	*P* value
Tumor location				
Central	15	5 (25%)	10 (50%)	0.06
Peripheral	25	15 (75%)	10 (50%)	
Lung disease				
COPD/bronchitis	4	2 (10%)	2 (10%)	0.51
None	36	18 (90%)	18 (90%)	
Diabetes				
No	28	14 (70%)	14 (70%)	0.58
Yes	12	6 (30%)	6 (30%)	
Histopathology				
Adenocarcinoma	23	13 (65%)	10 (50%)	0.39
Adenosquamous	1	1 (5%)		
Squamous cell carcinoma	15	6 (30%)	9 (45%)	
Highly undifferentiated	1		1 (5%)	
Age				
65 years or less	24	13 (65%)	11 (55%)	0.52
More than 65 years	16	7 (35%)	9 (45%)	
Smoking status				
Current smoker	21	12 (60%)	9 (45%)	0.63
Non-smoker	9	4 (20%)	5 (25%)	
Previous smoker	10	4 (20%)	6 (30%)	
Tumor size				
N/A	18	10	8	0.43
Size ≤ 5 cm	9	5 (50%)	4 (33.3%)	
Size > 5 cm	13	5 (50%)	8 (66.7%)	
SUVmax for lymph node				
N/A	22	14	8	0.00
SUVmax ≤ 3	8	6 (100%)	2 (16.7%)	
SUVmax > 3	10		10 (83.3%)	
SUVmax for primary tumor				
N/A	3	1	2	0.32
SUVmax ≤ 3	1	1 (5.3%)		
SUVmax > 3	36	18 (94.7%)	18 (100%)	

## Discussion

Our study has underlined the importance of multimodality staging of the MLNs in NSCLC. Furthermore, it has shown that ^18^F-FDG PET/CT had a reliable NPV which is comparable to that of EBUS/TBNA and mediastinoscopy, and higher than that of CT scan. As for true-positive and true-negative findings by ^18^F-FDG PET/CT, in our study, peripheral tumors constituted 78.9% of tumors deemed true-negative by ^18^F-FDG PET/CT. In addition, lymph nodes with SUVmax more than 3 were significantly more likely to be true-positive.

Over the last decade, ^18^F-FDG PET/CT has gained a wide acceptance in the staging of MLNs in NSCLC despite the observation that the number of original studies which have particularly investigated this important role in the staging of MLNs is not large enough when taking into consideration the significance of this topic. Our study has provided further knowledge about the strengths and limitations of PET/CT in this important clinical application. It has also compared the performance of ^18^F-FDG PET/CT and EBUS/TBNA in a considerable number of patients. Moreover, our retrospective real-life study has provided further evidence on the complementary role of the different modalities employed in the staging of MLNs in NSCLC with a relatively large cohort of patients. Such data may provide further direction to the multidisciplinary team of lung cancer in their everyday practice.

Our study is limited by virtue of its retrospective nature. In addition, only 57 patients were included in the main analyses from the original 101 patients given the histopathologic confirmation as the reference standard. Even then, not all lymph node stations were sampled, either by biopsy or lymph node dissection, hence a full analysis of all lymph node stations was not warranted, especially given the ethical and practical limitations of such an endeavor. The delineation of each lesion as true or false positive/negative was made based on the available histopathology of sampled LNs, follow-up imaging if performed, and subsequent MDC decisions.

Some studies have shown that ^18^F-FDG PET/CT has both higher sensitivity and higher specificity than CT scanning for the evaluation MLNs [7–9]. One study [8] reported that the sensitivity, specificity, PPV, NPV, and accuracy of ^18^F-FDG PET/CT in detecting hilar and MLN metastases were 74.2%, 73.2%, 54.4%, 86.8%, and 73.5%, respectively. A meta-analysis assessed the diagnostic value of ^18^F-FDG PET/CT in detecting metastatic lesions in NSCLC patients and pooled data from 56 studies involving 8,699 [9]. The pooled sensitivities and specificities of ^18^F-FDG PET/CT were 0.72 and 0.91 in determining MLN staging; 0.71 and 0.83 in intrathoracic staging; 0.78 and 0.90 in intrathoracic staging on a per-node basis.

Our results regarding the performance of ^18^F-FDG PET/CT have shown higher sensitivity but with a lower specificity when compared to published articles as shown thus far. This difference may be the result of following the qualitative visual approach in the assessment of metabolic status rather than the semi-quantitative (SUVmax.) approach that usually has been adopted in a considerable number of published articles, and presumably due to our cohort of patients. False positive and false negative results do occur on ^18^F-FDG PET/CT lung cancer staging, presumably due to the incapability of ^18^F-FDG PET/CT in differentiating uptake of lung cancer from that of infection/inflammation, and the spatial and contrast resolution limitations of ^18^F-FDG PET/CT, which would miss very small sites of metastatic disease [10].

Although the PPV of ^18^F-FDG PET/CT is poor, only 51.5% in our study, the NPV of ^18^F-FDG PET/CT is 100% in those with N1 staging and 82.6% in those with N2 and N3 staging. In patients where the ^18^F-FDG PET/CT achieved concordance with final N-stage, 21.6% underwent a change in management solely on the basis of ^18^F-FDG PET/CT findings.

Patients should undergo invasive nodal staging to exclude benign etiology of positive uptake and failure to do so would deny patients from potential curative resection. One study [11] evaluated the accuracy of ^18^F-FDG PET/CT in mediastinal staging compared with invasive mediastinal staging either by mediastinoscopy alone or by mediastinoscopy combined with thoracotomy. Based on ^18^F-FDG PET/CT alone, eight patients (out of 22; 36%) would have been denied potentially curative surgery if the mediastinal abnormalities detected by ^18^F-FDG PET/CT had not been evaluated with an invasive mediastinal procedure. Hence, pathologic confirmation of MLN abnormalities detected by ^18^F-FDG PET/CT is crucial. In our study, 13 patients (22.8%) had false-positive findings and would have been incorrectly upstaged by ^18^F-FDG PET/CT if this modality was solely relied upon in clinical management.

Gonzalez–Stawinski et al. prospectively compared the efficacy of ^18^F-FDG PET/CT to mediastinoscopy in 202 NSCLC cases. Out of 137 patients with negative PET findings, 16 (11.7%) were demonstrated to have N2 or N3 disease. Hence, the authors concluded that negative ^18^F-FDG PET/CT cannot exclude MLN involvement of lung cancer, and mediastinoscopy should be performed on every patient with pathologic confirmation [12]. Similar results were reported by Daniels et al. [13].

Multimodality staging of MLNs in NSCLC is of the utmost importance to lead to accurate staging and the proper management plan. In our cohort, there were four patients (7%) with false-negative findings and would have been incorrectly down-staged by ^18^F-FDG PET/CT if this modality was solely relied upon in clinical management. In three of these patients, there were no sizeable LNs, and ^18^F-FDG PET/CT and CT scan had missed metastatic lymphadenopathy that would have otherwise upstaged these patients. In the fourth patient, the ^18^F-FDG PET/CT quality was suboptimal due to patient noncompliance with the pre-scan medication regimen, and this led to false downstaging. Four patients also had false-negative mediastinoscopy findings (later confirmed by LN dissection) due to incomplete sampling during the intervention. There was only one false-negative finding by EBUS/TBNA, mainly due to the inherent drawback of the sampling procedure, where the tumor focus in the LN was missed and normal cells were extracted.

The 2014 European Society of Thoracic Surgeons algorithm for preoperative MLN staging updated the role of ^18^F-FDG PET/CT on NSCLC mediastinal staging as follows: (1) Direct surgery can be performed if all of the three criteria apply: No suspected lymph node on CT or PET, a tumor < 3 cm, and located in the outer third of the lung and (2) In case of enlarged node on CT or PET-positive nodes, tissue confirmation is indicated [14]. Our current results provide additional evidence to support this algorithm.

## Conclusion

Multimodality staging of the MLNs in NSCLC is essential to provide accurate staging and the appropriate treatment. ^18^F-FDG PET/CT has better overall diagnostic utility when compared to the CT scan. The NPV of ^18^F-FDG PET/CT in MLNs is reliable and comparable to the NPV of EBUS/TBNA. SUVmax of MLNs can help in predicting metastases, but nevertheless, a positive ^18^F-FDG PET/CT MLNs particularly if such a result would change the treatment plan, should be verified histopathologically.

## Supplementary Information

**Additional file 1: **Performance of the different modalities according to lymph node station in patients with histopathological confirmation.

**Additional file 2: **Performance of the different modalities according to N-stage categories.

## Data Availability

The datasets during and/or analyzed during the current study available from the corresponding author on reasonable request.

## Abbreviations

NSCLC: Non-small-cell lung cancer
^18^F-FDG PET: [Fluorine-18] fluoro-2-deoxy-D-glucose positron emission tomography
PET/CT: Positron emission tomography/computed tomography
CT: Computed tomography
EBUS/TBNA: Endobronchial ultrasound/transbronchial needle aspirate
MLNs: Mediastinal lymph nodes
MLN: Mediastinal lymph node
LN: Lymph node
N0: No lymph node metastases
N1: Ipsilateral hilar lymph
N2: Ipsilateral mediastinal lymph metastases
N3: Contralateral mediastinal lymph metastases
IRB: Institutional Review Board
KHCC: King Hussein Cancer Center
MDC: Multidisciplinary clinic
MIP: Maximum intensity projection
ROSE: Rapid on-site evaluation was performed when available
NPV: Negative predictive value
PPV: Positive predictive value
MBq: Megabecquerel
SUVmax: Maximal standardized uptake value
RUL: Right upper lobe
LUL: Left upper lobe
