# Supplementary material for: Impact of 18F-FDG PET/CT, CT and EBUS/TBNA on preoperative mediastinal nodal staging of NSCLC
Source: BMC Med Imaging. 2021 Mar 17;21:49. doi: 10.1186/s12880-021-00580-w (PMC7967993; doi:10.1186/s12880-021-00580-w)
Supplement: Supplementary file 1 — Additional file 1: Performance of the different modalities according to lymph node station in patients with histopathological confirmation. [file 12880_2021_580_MOESM1_ESM.docx]

| Modality | ^18^F-FDG PET/CT | | | | | | CT scan | | | | | | | EBUS | | Mediastinoscopy | | |
| --- | --- | --- | --- | --- | --- | --- | --- | --- | --- | --- | --- | --- | --- | --- | --- | --- | --- | --- |
| LN station | **2** | **4** | **5** | **7** | **9** | **10** | **2** | **4** | **5** | | **7** | **9** | **10** | **4** | **7** | **4** | **7** |  |
| Sensitivity  95% CI | 100  2.50 - 100.00 | 75  34.91 - 96.81 | - | 50  11.81 - 88.19 | - | 75  42.81 - 94.51 | - | 85.7  42.13 - 99.64 | | - | 16.7  0.42 - 64.12 | - | 63.6  30.79 - 89.07 | 100  29.24 - 100.00 | 100  29.24 - 100.00 | 66.7  9.43 - 99.16 | 33.3  0.84 - 90.57 |  |
| Specificity  95% CI | 93.6  78.58 - 99.21 | 82.1  66.47 - 92.46 | 92.9  66.13 - 99.82 | 79.6  64.70 - 90.20 | 100  83.89 - 100 | 55.2  35.69 - 73.55 | 80.7  62.53 - 92.55 | 71.8  55.13 - 85.00 | | 78.6  49.20 - 95.34 | 70.5  54.80 - 83.24 | 100  83.89 - 100.00 | 44.8  26.45 - 64.31 | 66.7  9.43 - 99.16 | 100  63.06 - 100.00 | 96.9  83.78 - 99.92 | 100  89.11 - 100.00 |  |
| PPV  95% CI | 33.3  11.57 - 65.64 | 46.2  28.18 - 65.18 | - | 25  11.02 - 47.28 | - | 40.9  29.17 - 53.78 | - | 35.3  23.31 - 49.47 | | - | 7.1  1.20 - 32.77 | - | 30.4  20.09 - 43.23 | 75  37.71 - 93.70 | 100  NA | 66.7  19.86 - 94.17 | 100  NA |  |
| NPV  95% CI | 100  NA | 94.1  82.68 - 98.17 | 92.9  91.83 - 93.76 | 92.1  83.79 - 96.34 | 91.3  91.30 - 91.30 | 84.2  65.49 - 93.75 | 96.2  95.46 - 96.74 | 96.6  81.86 - 99.43 | | 91.7  89.32 - 93.53 | 86.1  80.51 - 90.29 | 91.3  91.30 - 91.30 | 76.5  57.41 - 88.68 | 100  NA | 100  NA | 96.9  86.21 - 99.35 | 94.1  87.79 - 97.27 |  |
| Accuracy  95% CI | 93.8  79.19 - 99.23 | 80.9  66.74 - 90.85 | 86.7  59.54 - 98.34 | 76  61.83 - 86.94 | 91. 71.96 - 98.93 | 61  44.50 - 75.80 | 78.1  60.03 - 90.72 | 73.9  58.87 - 85.73 | | 73.3  44.90 - 92.21 | 64  49.19 - 77.08 | 91.3  71.96 - 98.93 | 50  33.80 - 66.20 | 83.3  35.88 - 99.58 | 100  71.51 - 100.00 | 94.3  80.84 - 99.30 | 94.3  80.84 - 99.30 |  |

Table S1: Diagnostic utility of the different imaging modalities per-lymph-node-station analysis for concordance with final his-pathological diagnosis was performed on 57 patients with nodal tissue confirmation. In comparison - CT, ^18^F-FDG PET/CT had better specificity, NPV and accuracy in detecting metastatic lesions in lymph nodes from stations 2, 4, 5, 7, 9 and 10. Stations 4 and 7 were also assessed in EBUS and mediastinoscopy findings. In station 4, ^18^F-FDG PET/CT had higher specificity (82.1%) than CT scan (71.8%) and EBUS/TBNA (66.7%) but was superseded by mediastinoscopy (96.9%). In station 7, mediastinoscopy and EBUS/TBNA had higher specificity (100% for both) and accuracy (94.3% and 100%, respectively) compared - ^18^F-FDG PET/CT (specificity 79.6% and accuracy 76%) and CT scan (specificity 70.5% and accuracy 64%).
