# Supplementary material for: Impact of 18F-FDG PET/CT, CT and EBUS/TBNA on preoperative mediastinal nodal staging of NSCLC
Source: BMC Med Imaging. 2021 Mar 17;21:49. doi: 10.1186/s12880-021-00580-w (PMC7967993; doi:10.1186/s12880-021-00580-w)
Supplement: Supplementary file 2 — Additional file 2: Performance of the different modalities according to N-stage categories. [file 12880_2021_580_MOESM2_ESM.docx]

Patients with N1 stage confirmed with histopathology

Patients with N1 final staging were included in this analysis. In comparison to CT, ^18^F-FDG PET/CT had a better sensitivity (100% vs. 50%), specificity (60.6% vs. 38.2%), NPV (100% vs. 81.3%) and accuracy (67.5% vs. 40%). Specificity and accuracy of mediastinoscopy (100%) and EBUS/TBNA (90.9% and 91.7%, respectively) superseded those of ^18^F-FDG PET/CT (60.6% and 67.5%, respectively). A per-lymph-node-station analysis reveals improved specificity of ^18^F-FDG PET/CT compared to CT in stations 2, 4, 5 and 7, with equal values (100%) in station 9.

**Patients with N2 and N3 stage confirmed with histopathology**

Patients with N2 and N3 final staging were included in this analysis. In comparison to CT, ^18^F-FDG PET/CT had a better sensitivity (76.5% vs 56.3%), specificity (67.9% vs. 50%), NPV (82.6% vs. 66.7%) and accuracy (71.1% vs. 52.3%). ^18^F-FDG PET/CT also superseded mediastinoscopy in sensitivity (76.5% vs. 33.3%) with a slightly higher NPV (82.6% vs. 81%). EBUS/TBNA, however, and in comparison, to ^18^F-FDG PET/CT, had better sensitivity (80% vs. 76.5%), specificity (85.7% vs. 67.9%), NPV (85.7% vs. 82.6%) and accuracy (83.3% vs. 71.1%).

A per-lymph-node-station analysis reveals improved specificity, NPV and accuracy of ^18^F-FDG PET/CT compared to CT in stations 2, 4, 7 and 10, the only exceptions being higher NPV (95.5% vs. 92.6%) and accuracy (76.5% vs. 71%) for CT in station 4 compared to ^18^F-FDG PET/CT.
